# A straightforward approach utilizing an exponential model to compensate for turbidity in chemical oxygen demand measurements using UV-vis spectrometry

**DOI:** 10.3389/fmicb.2023.1224207

**Published:** 2023-07-10

**Authors:** Hongliang Wang, Houkui Xiang, Tongqiang Xiong, Jinping Feng, Jianquan Zhang, Xuemei Li

**Affiliations:** ^1^School of Automation, Hubei University of Science and Technology, Xianning, China; ^2^Office of Laboratory Management and Teaching Facilities Development, Renmin University of China, Beijing, China

**Keywords:** chemical oxygen demand, turbidity compensation, ultraviolet-visible spectrometry, turbid water, exponential model

## Abstract

Recently, ultraviolet-visible (UV-vis) absorption spectrometry has garnered considerable attention because it enables real-time and unpolluted detection of chemical oxygen demand (COD) and plays a crucial role in the early warning of emerging organic contaminants. However, the accuracy of detection is inevitably constrained by the co-absorption of organic pollutants and turbidity at UV wavelengths. To ensure accurate detection of COD, it is necessary to directly subtract the absorbance caused by turbidity from the overlaid spectrum using the principle of superposition. The absorbance of COD is confined to the UV range, whereas that of turbidity extends across the entire UV-vis spectrum. Therefore, based on its visible absorbance, the UV absorbance of turbidity can be predicted. In this way, the compensation for turbidity is achieved by subtracting the predicted absorbance from the overlaid spectrum. Herein, a straightforward yet robust exponential model was employed based on this principle to predict the corresponding absorbance of turbidity at UV wavelengths. The model was used to analyze the overlaid absorption spectra of synthetic water samples containing COD and turbidity. The partial least squares (PLS) method was employed to predict the COD concentrations in synthetic water samples based on the compensated spectra, and the corresponding root mean square error (RMSE) values were recorded. The results indicated that the processed spectra yielded a considerably lower RMSE value (9.51) than the unprocessed spectra (29.9). Furthermore, the exponential model outperformed existing turbidity compensation models, including the Lambert-Beer law-based model (RMSE = 12.53) and multiple-scattering cluster method (RMSE = 79.34). Several wastewater samples were also analyzed to evaluate the applicability of the exponential model to natural water. UV analysis yielded undesirable results owing to filtration procedures. However, the consistency between the compensated spectra and filtered wastewater samples in the visible range demonstrated that the model is applicable to natural water. Therefore, this proposed method is advantageous for improving the accuracy of COD measurement in turbid water.

## 1. Introduction

With the rapid development of industries and explosive population growth, considerable amounts of emerging organic contaminants are being discharged into natural water bodies, causing severe environmental issues in the world today (Lapworth et al., 2012; Pal et al., 2014). Chemical oxygen demand (COD), which represents the level of organic pollutants in water, is a crucial indicator of the impact of emerging organic contaminants on water quality. Having reliable COD analysis methods is essential for effective water quality monitoring in wastewater treatment, a prerequisite for controlling water pollution. Currently, methods for measuring COD include the potassium permanganate method, the dichromate method, the flow-injection analysis method, the electrochemical analysis method, and ultraviolet-visible (UV-vis) spectroscopy (Li et al., 2018). Among these methods, UV-vis spectroscopy is the most commonly used optical technique for assessing the COD concentration in water (Hu and Wang, 2017; Xu et al., 2023). This approach indirectly measures the absorption of UV-vis light by organic matter to determine COD levels.

UV-vis spectroscopy is widely utilized for COD measurement due to its rapid measurement time, lack of secondary pollution, reagent-free nature, and ability to provide real-time determination (Carré et al., 2017; Ma et al., 2018). However, UV-vis spectroscopy is typically limited by the interference of suspended particles; thus, effective methods must be adopted to suppress the influence of interfering factors and improve prediction accuracy (Wu et al., 2017; Chen et al., 2021). Turbidity compensation has become a research focus in the area of COD measurement based on UV-vis spectroscopy (Hu et al., 2016; Li et al., 2019; Hu et al., 2020; Kang et al., 2022; Zhang et al., 2022). Nevertheless, the turbidity compensation algorithm for COD measurement has not been extensively researched.

Presently, there are primarily two categories of turbidity compensation methods (Li et al., 2019). In one category, the COD value is corrected by using a model of the COD prediction error versus turbidity; however, such a model cannot completely eliminate the impact of turbidity, leading to poor versatility. In the second category, the absorption spectrum is determined by subtracting the absorbance caused by turbidity, requiring a complex model to calculate the turbidity absorbance. Apart from exhibiting large deviations, these complex models pose challenges for embedded programming and local calibration, rendering them unsuitable for online sensors. To address these limitations, the main objectives of this study were as follows: (1) develop an effective turbidity compensation method for COD measurement using UV-vis spectrometry, (2) investigate the applicability of embedded programming and local calibration as well as the possibility of online spectrometers to the model, (3) evaluate the accuracy of COD prediction in mixtures containing COD and turbidity, and (4) explore the feasibility of measuring COD in natural water using this model.

## 2. Materials and methods

### 2.1. Experimental device

**Figure 1:**
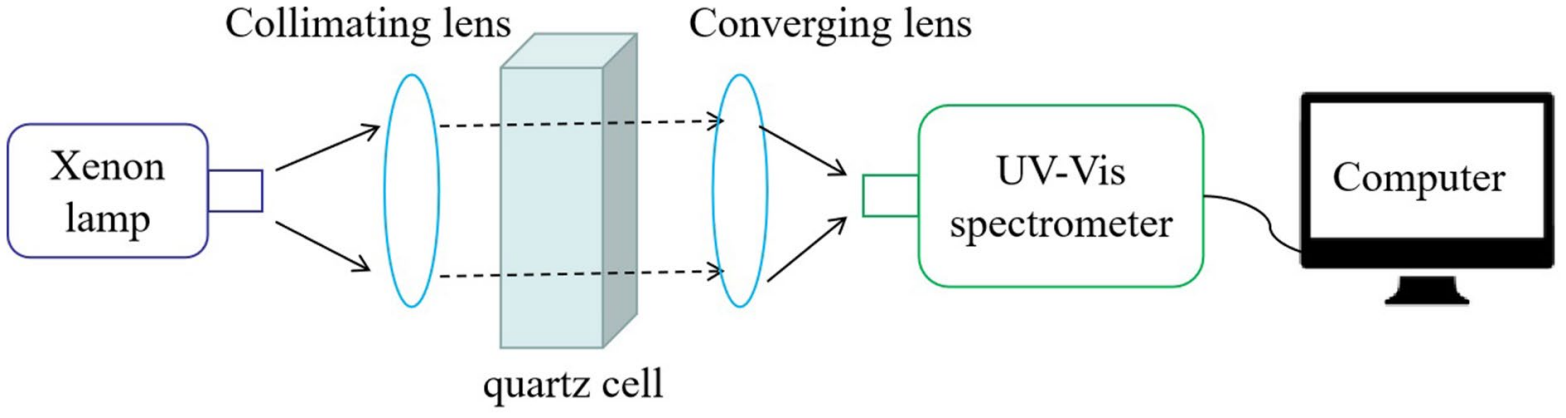
Schematic illustration of spectral acquisition system.

The spectral measuring system primarily comprises four parts: a light source, quartz cell, micro UV-vis spectrometer, and computer (Figure 1). The xenon lamp source (42 mm × 42 mm × 37 mm, L13651-01, HAMAMATSU) produced a light of 185 to 2000 nm, which was collimated by a lens and passed through the quartz cell. The attenuated light emanating from the quartz cell was gathered by a 256-pixel microspectrometer (15 mm × 22 mm × 35 mm, UV20 UV-vis, Horiba) through a converging lens. The operation and data collection of the spectrometer were controlled using an additional computer. The xenon lamp and spectrometer used in this study were compact enough to be enclosed within a cylinder, enabling the development of streamlined spectral probes. Ensuring that the optical system exhibits sufficient stability is crucial, because irregular factors can easily disrupt the absorption law of turbidity across the entire spectrum.

### 2.2. Water samples and materials

The absorption behavior of turbidity was investigated using formazine owing to its excellent optical stability. The formazine suspensions were prepared via a chemical reaction between hydrazine sulfate and hexamethylenetetramine, following the method outlined in ISO 7027-2016. To establish a mathematical model for turbidity compensation, 15 formazine suspensions of 10–200 FNU (formazine nephelometric units) were prepared. Further, a series of mixtures containing COD and turbidity were measured to validate the performance of the established model. Subsequently, 30 different mixtures with specific concentrations of COD and turbidity were prepared using a COD stock solution and formazine suspension. The COD stock solution was prepared by dissolving potassium hydrogen phthalate in water. All reagents were purchased from Maclin (China), and all solutions were prepared using ultrapure water (18.2 MΩ cm at 25°C) obtained from a Millipore Milli-Q system.

Several wastewater samples were also analyzed to assess the applicability of the model to natural water. The samples were collected at the inlet of an A/A/O municipal wastewater treatment system located in the Low Carbon Water Environmental Technology Center at Renmin University in China. The turbidity absorbance of wastewater was determined by calculating the difference spectra between filtered and unfiltered samples, with the filtration process utilizing 0.45 μm membranes (Haining Kewel Company, China). Finally, the feasibility of the model was assessed through a comparison of the compensated and measured spectra from the filtered water samples.

### 2.3. Measurement procedures

All water samples were analyzed using the experimental apparatus depicted in Figure 1, which employs a single optical path design and utilizes a quartz cell with an optical path length of 10 mm. To ensure high stability in acquiring spectra, the integration time and average signal of the spectrometer were set to 2000 ms and three times, respectively. Prior to measurement, all water samples underwent thorough agitation through rapid particle precipitation. Each water sample was measured five times and the obtained spectra were averaged to reduce the influence of random errors. The transmitted spectrum of the ultrapure water was used as a reference to calculate the sample absorbance.

## 3. Results and discussion

### 3.1. UV-vis absorption behavior of turbidity and proposed exponential model

**Figure 2:**
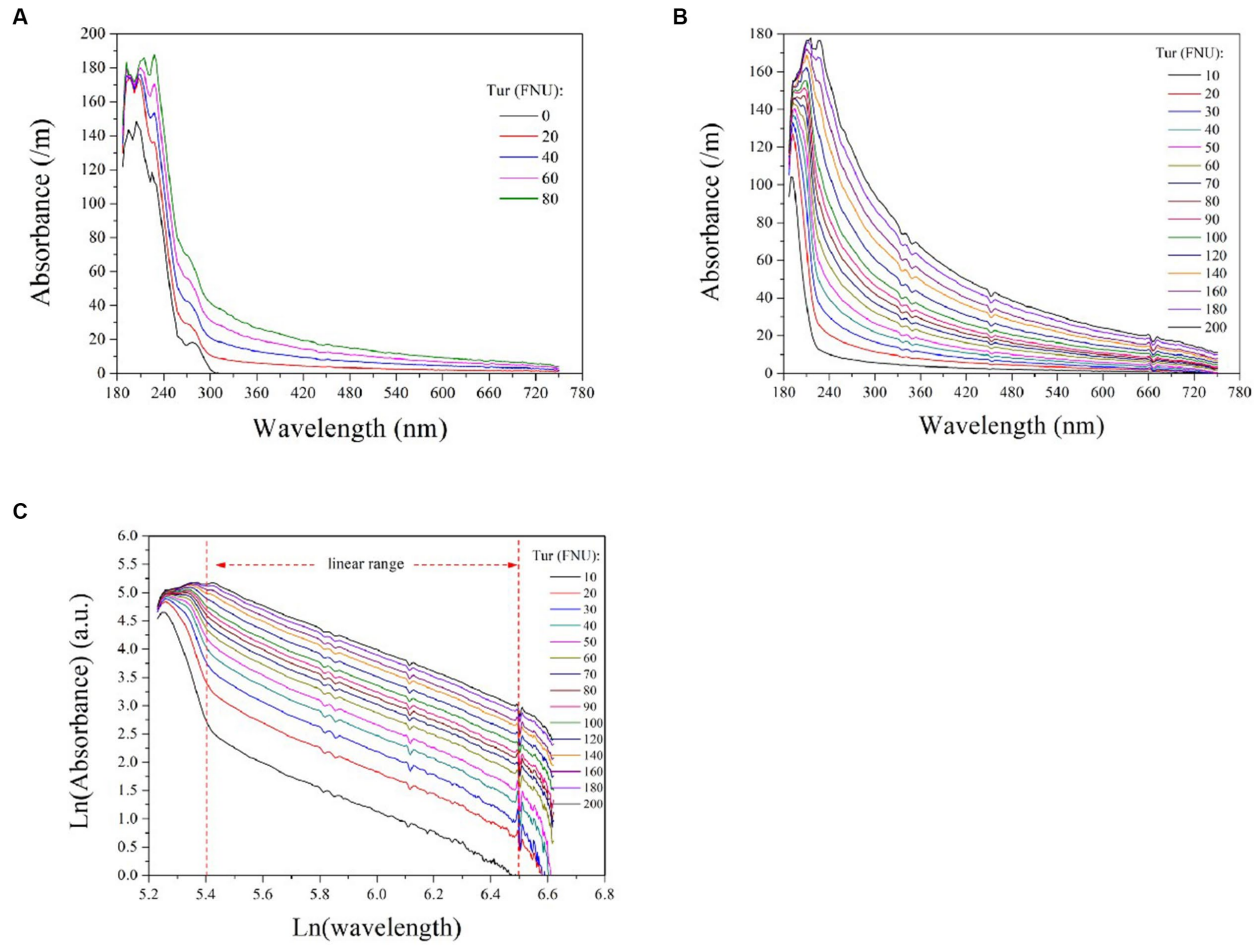
The absorption spectra of 50 mg/L COD solutions mixed with varying turbidity concentrations **(A)**; the absorption spectra of formazine suspensions at different concentrations **(B)**; the logarithmic UV-vis spectra of formazine suspensions **(C)**. The two vertical dashed lines indicate the linear range of the curves, which is between 5.4 < Ln (wavelength) <6.5 or 220 nm < wavelength < 660 nm.

To investigate the impact of turbidity on COD detection via UV-vis spectrometry, we measured the absorption spectra of 50 mg/L COD solutions mixed with varying concentrations of turbidity, as depicted in Figure 2A. Absorption by the pure COD solution occurred predominantly within the UV range (180 ~ 320 nm) and increased with increasing turbidity concentration. This confirms that water turbidity poses a significant challenge to measuring COD using UV-vis spectrometry. Therefore, it is imperative to establish an efficient model for predicting the equivalent absorbance of turbidity at each UV wavelength to obtain the absorbance of COD alone by subtracting the overlapping spectrum. This method enables accurate detection of COD through the compensated ultraviolet spectrum.

To establish a model for predicting the equivalent absorbance of turbidity at different wavelengths, further investigation into its absorption behavior across the entire spectrum is required. For this purpose, we measured the absorbance of formazine suspensions with varying concentrations ranging from 10 to 200 FNU and presented the resulting spectra in Figure 2B. Turbidity exhibits absorbance over the entire spectrum owing to light scattering caused by suspended particles, with higher turbidity corresponding to stronger absorbance at each wavelength. For each absorption spectrum, the absorbance decreases rapidly initially and then gradually with longer wavelengths. Several studies have reported an exponential increase in particle absorbance at shorter wavelengths, which is visually consistent with the absorption spectra of formazine suspensions (Langergraber et al., 2003; Berho et al., 2004). We hypothesized that an exponential model could be utilized to predict the absorbance of turbidity at each wavelength, enabling effective turbidity compensation.

Based on the aforementioned hypothesis, the logarithmic UV-vis spectra of the formazine suspensions were transformed to linear form, as illustrated in Figure 2C. It is noteworthy that the logarithmic spectra exhibit a visually linear trend within the range of 220 to 660 nm (5.4 < Ln (wavelength) < 6.5). The logarithmic spectra were subjected to linear regression analysis, with the resulting data presented in Table 1. The high values of *R*^2^ (>0.98) indicate a strong linear correlation between the variables under investigation. However, there are nonlinear regions within the logarithmic spectra, particularly at shorter and longer wavelengths. The nonlinearity observed at shorter wavelengths (<220 nm) may be attributed to the presence of co-absorptive substances, such as organic compounds and nitrates. As absorbances at longer wavelengths (>660 nm) are relatively small, they are susceptible to random noise interference, causing nonlinearity. In summary, the relationship between turbidity absorbance and wavelength can be expressed by Eq. (1) below:

(1) $$Ln\left(A_{Tur}\right)=k\cdot Ln\left(\lambda \right)+b$$

**Table 1:** The results of linear regression analysis on the logarithmic spectra of formazine suspensions.

Turbidity (FNU)	Slope *k*	Intercept *b*	*R* ^2^
10	−2.13	2.21	0.988
20	−2.16	2.92	0.995
30	−2.10	3.35	0.993
40	−2.12	3.59	0.995
50	−2.10	3.77	0.996
60	−2.09	3.93	0.997
70	−2.07	4.06	0.997
80	−2.09	4.19	0.998
90	−2.10	4.30	0.998
100	−2.02	4.37	0.998
120	−2.03	4.54	0.998
140	−2.04	4.70	0.998
160	−2.06	4.81	0.998
180	−2.01	4.91	0.998
200	−2.00	4.99	0.998

The absorbance of turbidity at wavelength *λ* is denoted by *A_Tur_*, and *k* and *b* represent the slope and intercept of the logarithmic spectra, respectively. However, the linearity of Equation (1) may be affected by the stability of the experimental system, with an unstable system often resulting in a downward trend in the logarithmic spectra. The instability of the system is primarily attributed to the impact of the spectrometer and xenon lamp. Specifically, the highly sensitive charge-coupled device detector in the spectrometer is susceptible to noise, ambient light, and dark-current interference. In contrast, the spectrometer utilized in this study was equipped with a less sensitive complementary metal-oxide-semiconductor detector (e.g., UV20, Horiba), which tends to be less susceptible to these impact factors. Additionally, the light intensity of the xenon lamp must be sufficiently strong to ensure a high signal-to-noise ratio in the system.

**Figure 3:**
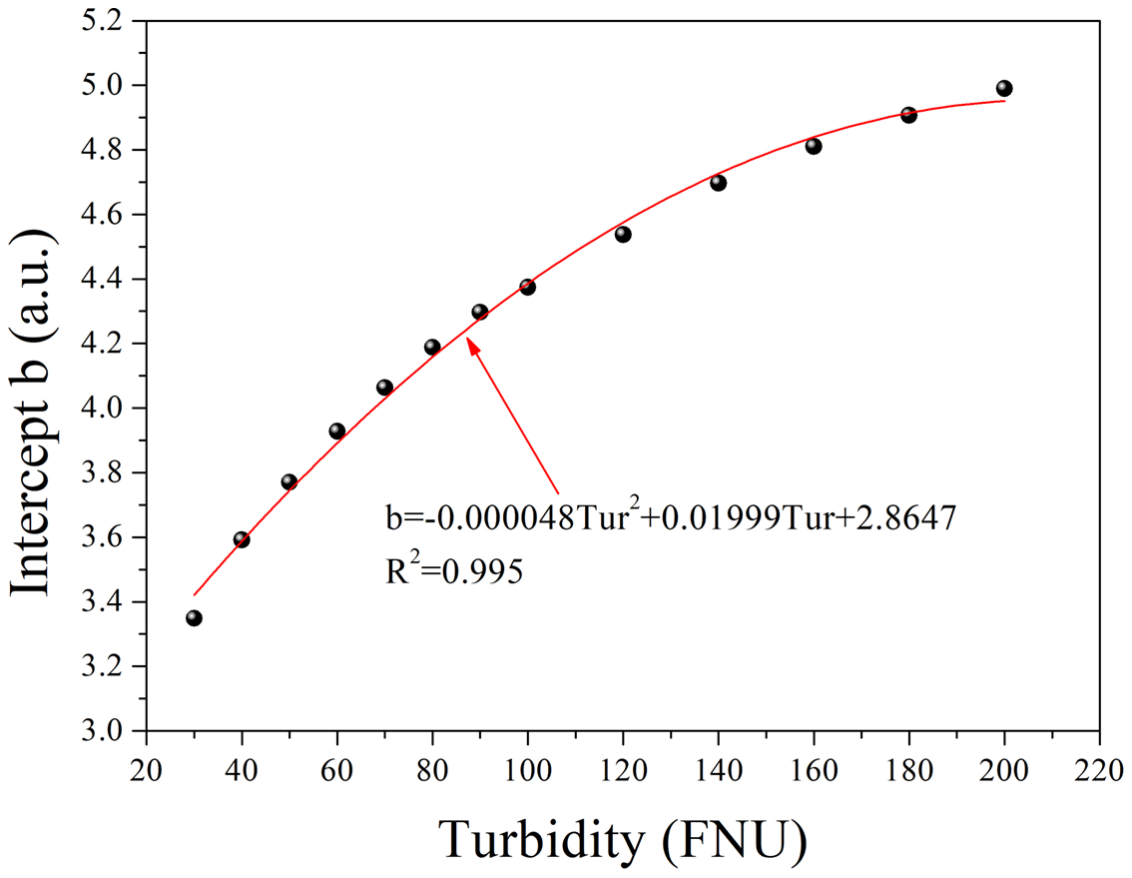
The relationship between intercept *b* and the concentration of turbidity.

Table 1 clearly shows that parameter b gradually increases with increasing turbidity, while parameter k remains relatively constant. Furthermore, an investigation into the relationship between parameter b and turbidity was conducted, as depicted in Figure 3. To simplify the local calibration, a polynomial function was utilized to fit the curve with an *R*^2^ value of 0.995. A quadratic relationship between parameter b and turbidity was demonstrated, allowing for the determination of parameter b as a function of turbidity based on Eq. (2) as follows:


(2) $$b=m\cdot T{\mathrm{ur}}^{2}+n\cdot Tur+p$$

The quadratic function is represented by the coefficients *m*, *n*, and *p*, while *Tur* denotes the turbidity concentration. Substituting Eq. (2) into Eq. (1) allows the prediction of the absorbance of any given turbidity concentration at each wavelength using Eq. (3):


(3) $$A_{Tur}=\exp\left(m\cdot Tur^{2}+n\cdot Tur+p\right)\cdot \lambda^{k}$$

By applying the superposition principle between COD absorbance and equivalent turbidity absorbance, compensation of the entire UV-vis spectrum can be achieved by subtracting the equivalent turbidity absorbance. Subsequently, the absorbance of COD alone can be obtained using Eq. 4 as follows:


(4) $$A_{COD}=A_{raw}-A_{Tur}=A_{raw}-\exp\left(m\cdot Tur^{2}+n\cdot Tur+p\right)\cdot \lambda^{k}$$

where *A_raw_* represents the absorbance of the raw spectrum at a specific wavelength *λ* and A_COD_ denotes the compensated absorbance of COD at the same wavelength. The latter can be transformed into a COD concentration by utilizing multivariate analysis tools. Measuring the turbidity prior to measuring COD is recommended because COD absorbance occurs in the UV range rather than in the visible range of the spectrum (Figure 2A). Turbidity can be measured by utilizing the absorbance at visible wavelengths, such as the wavelength band of 500 to 750 nm (Fleischmann et al., 2002). As depicted in Figure 2B, a proportional relationship exists between turbidity and the absorbance of visible wavelengths; thus, multivariate analysis tools (e.g., partial least squares method; PLS) can also be employed for turbidity measurement.

### 3.2. Validation of the proposed exponential model

#### 3.2.1. Chemical oxygen demand prediction by exponential model and its comparison with the Lambert-Beer law and multiple-scattering cluster method

**Figure 4:**
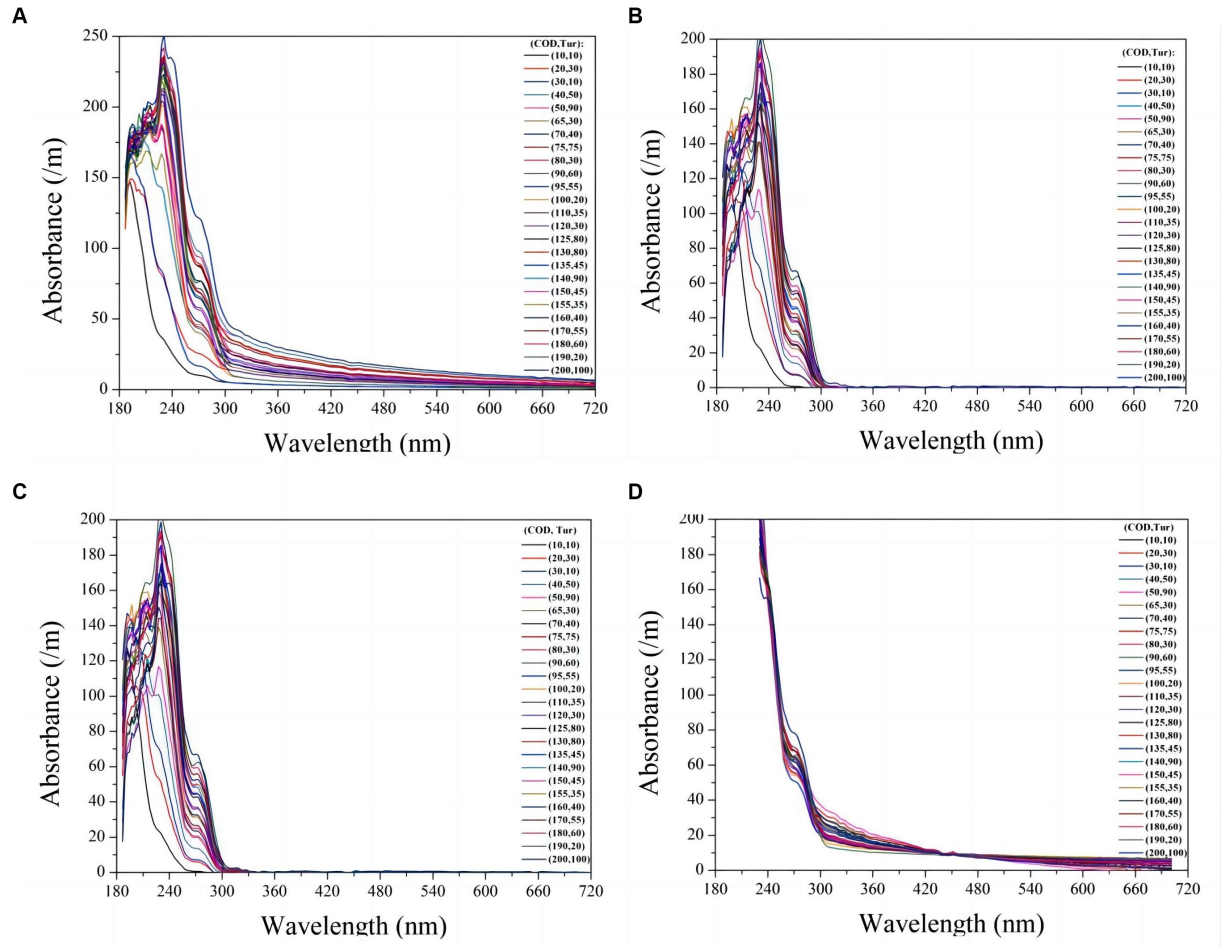
The raw UV-vis spectra of 30 mixtures containing COD and turbidity are presented **(A)**. The compensated spectra of the mixtures using exponential model, LBL, and MSC model are shown in **(B)**, **(C)**, and **(D)** respectively.

To verify the compensation performance of the exponential model, a series of mixtures containing COD and turbidity were measured, and the corresponding raw spectra are presented in Figure 4A. Subsequently, these raw spectra underwent processing using Eq. (4), resulting in compensated absorption spectra, as shown in Figure 4B. Other turbidity compensation models, such as the Lambert-Beer law-based (LBL) and multiple-scattering cluster (MSC) models mentioned in the literature, were also examined for comparison with the exponential model presented here (Wu et al., 2017; Li et al., 2019; Chen et al., 2021). The compensated spectra from the LBL and MSC models are shown in Figures 4C,D, respectively. The compensated spectra of MSC exhibit distinct deviations from those of the exponential and LBL models. Specifically, after compensation by the MSC model, the absorbance of water samples at visible wavelengths remains nonzero, whereas it approaches zero in the compensated spectra of the LBL and exponential models. This discrepancy can be attributed to turbidity in the water samples, which the MSC model cannot effectively eliminate. Moreover, the majority of the UV spectra tend to cluster together in the MSC model, which disrupts the proportional relationship between UV absorption and COD concentration, leading to decreasing COD measurement accuracy. In contrast, the LBL and exponential models provide highly dispersed UV spectra that enable more accurate predictions of COD.

**Figure 5:**
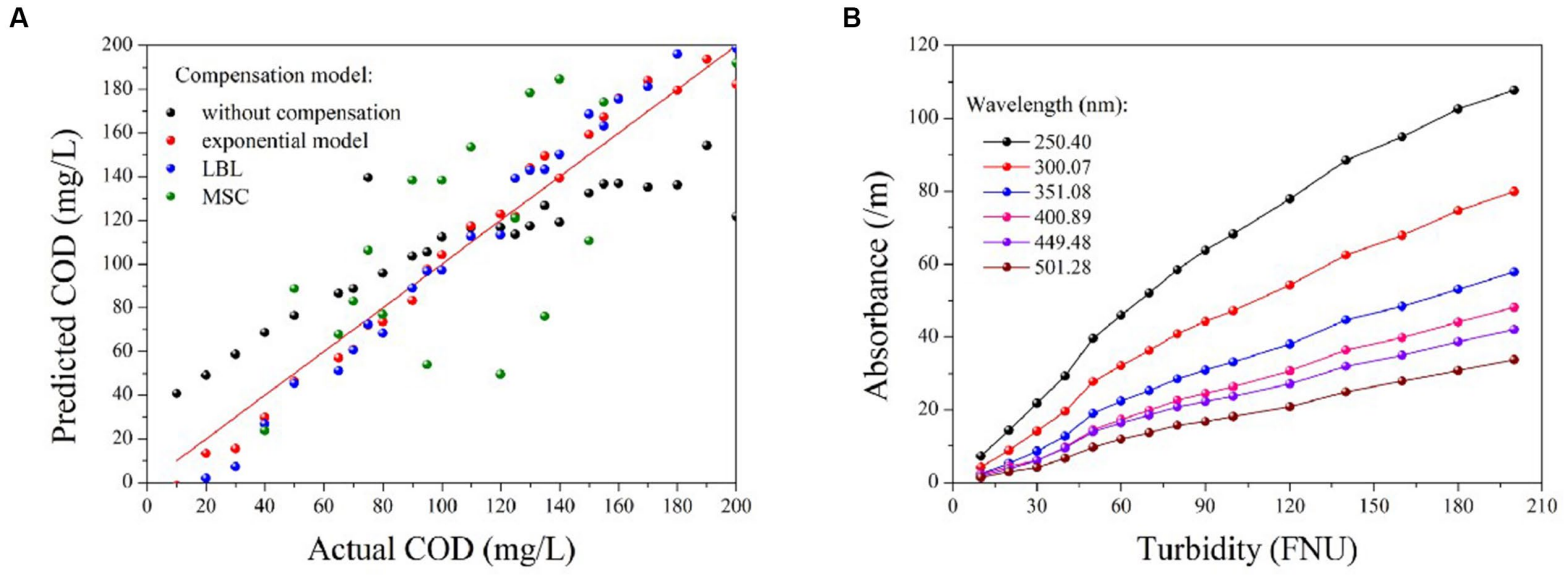
The prediction results of COD based on the compensated spectra provided by different methods **(A)**; the correlation between turbidity and absorbance at various wavelengths **(B)**.

The PLS method has been demonstrated to provide the most robust multivariate calibrations for addressing concentration-spectra relationships. As such, it is employed herein to retrieve the COD concentration based on the compensated absorption spectra (Wold et al., 2001; Lepot et al., 2016; Li et al., 2020). Figure 5A displays the predictions of the PLS models based on four types of compensated spectra, with their corresponding *R*^2^ and RMSE values listed in Table 2. The results obtained from the LBL and exponential models are generally consistent with the actual COD values, exhibiting small RMSE values. In contrast, the MSC predictions deviate greatly from the actual values, resulting in larger RMSE values. Therefore, the MSC model is comparatively less effective in compensating for turbidity than the exponential and LBL models.

**Table 2:** The predictive outcomes of PLS models based on diverse compensated spectra.

Compensation method	Without compensation	Exponential model	LBL	MSC
*R* ^2^	0.79	0.98	0.98	0.72
RMSE	29.90	9.51	12.53	79.34

Although the accuracy of the COD prediction in turbid water was significantly enhanced by the LBL and exponential models, a lower RMSE value was achieved only with the exponential model. This outcome may be attributed to the LBL model employing Lambert-Beer’s law to simulate the turbidity absorbance at each wavelength. It is well known that the Lambert-Beer law establishes a linear correlation between absorbance and solute concentration. However, this linearity is only observed at low concentrations at which the absorbance of the solute remains below a certain threshold. As previously stated, the absorbance of the turbidity increases exponentially as the wavelength decreases. Therefore, the relationship between absorbance and turbidity becomes less linear at shorter wavelengths, as shown in Figure 5B. Owing to this nonlinearity in the UV region, the LBL method performs worse in the UV than in the visible region. In contrast, the turbidity absorption conforms almost entirely to the exponential law across the entire spectrum. Therefore, the performance of the exponential model is consistent from the UV to the visible regions. As UV absorption provides more informative data for COD prediction, the RMSE value obtained by the exponential model was lower than that obtained with the LBL model.

#### 3.2.2. Verification of the COD measurement in natural water using the exponential model

**Figure 6:**
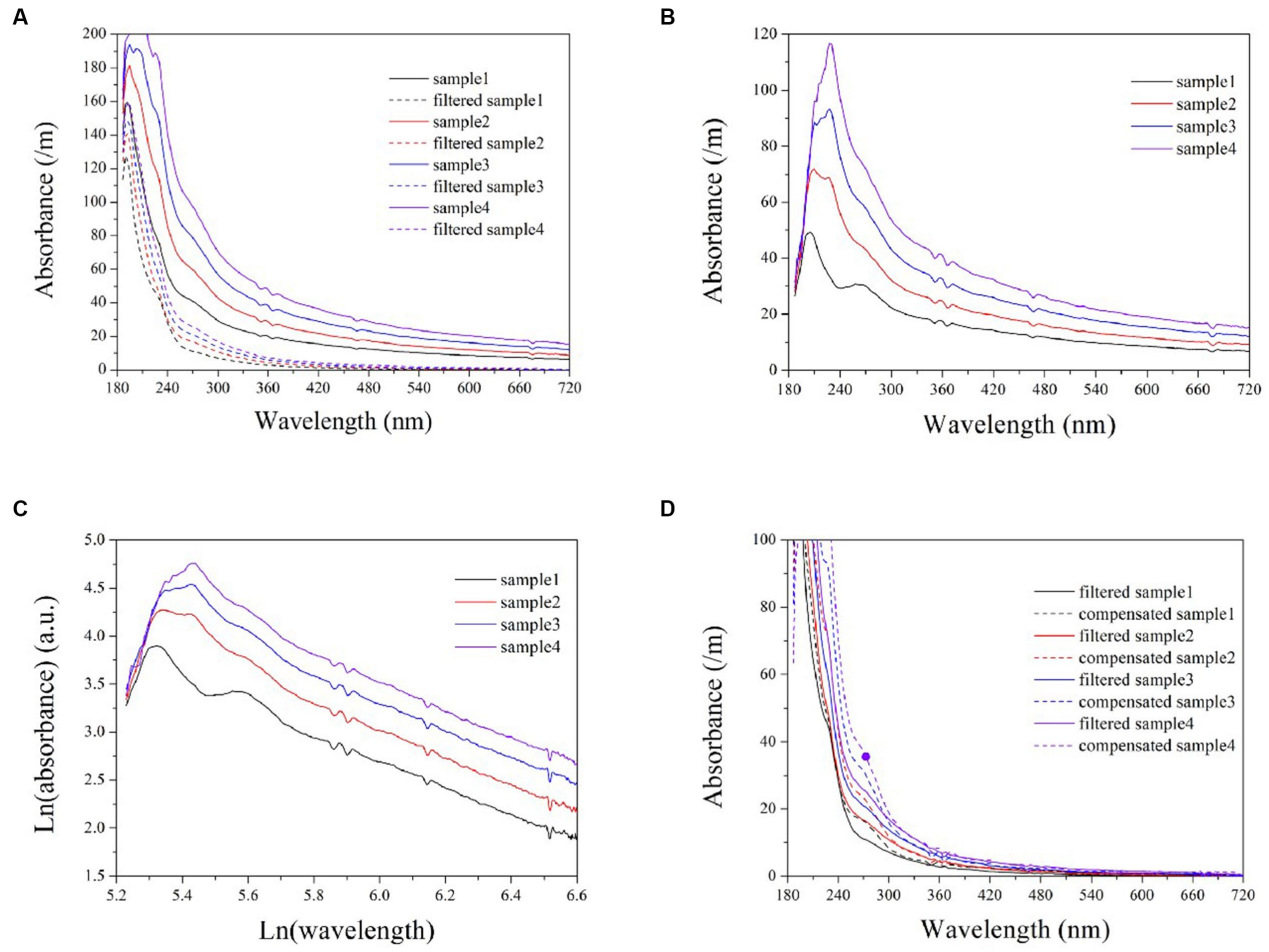
The UV-vis spectra of wastewater samples, both filtered and unfiltered **(A)**; turbidity spectra in wastewater obtained by calculating the difference between filtered and unfiltered samples **(B)**; logarithmic turbidity spectra in wastewater samples **(C)**; a comparison between compensated spectra and filtered sample spectra **(D)**.

Turbidity in natural water is caused by the presence of numerous particles of varying properties, resulting in multiple scattering (Huber and Frost, 1998). To assess the applicability of the exponential model in natural water, several wastewater samples were also analyzed. The turbidity absorption in wastewater can be determined by calculating the difference spectra between filtered and unfiltered samples, as illustrated in plots (A) and (B) of Figure 6. Subsequently, the logarithmic spectra of turbidity can be obtained, as shown in Figure 6C. Here, the logarithmic spectra exhibit a visually linear and uniform trend at ln(wavelength) >5.8 (wavelength > 320 nm). An exponential model can be established based on the linear portions of the spectra to predict the equivalent absorbance of turbidity at each wavelength. The elimination of cross-sensitivity in the UV range is achieved by subtracting the equivalent absorbance. By comparing Figures 2C, 6C, it is evident that the logarithmic spectra slopes of formazine suspensions exhibits a significantly greater magnitude than that of turbidity in wastewater. The disparity in particle size between the formazine suspension and wastewater turbidity is the primary cause of this phenomenon. Several studies have indicated that particulates with smaller diameters tend to exhibit a steep absorption spectrum, whereas those with larger diameters typically display a more gradual absorption spectrum (Huber and Frost, 1998; Berho et al., 2004). Therefore, the smaller particle size of formazine particles results in a steeper logarithmic spectrum compared to wastewater turbidity.

Artificial filtration can also eliminate the cross-sensitivity of turbidity, allowing for a visual evaluation of the performance of the exponential model through comparison with filtered samples, as shown in Figure 6D. The compensated spectra are highly consistent with the filtered spectra within the wavelength band of 320 ~ 720 nm. However, the compensated spectra exhibit higher values than the filtered spectra at the 200 ~ 320 nm range, potentially resulting in positive deviations during the COD measurements.

Although the compensated spectra exhibited inconsistencies with those of the filtered samples in the UV region, these discrepancies are primarily attributable to the filtration process itself, which results in a loss of COD and subsequently lower filtered spectra in this wavelength range. Therefore, it can be concluded that the deviation observed within the UV range is not due to any shortcomings inherent within the exponential model. In achieving a high level of consistency between the compensated spectra and the filtered spectra in the visible range, the exponential model demonstrates that it remains applicable for simulating turbidity absorption behavior in natural water. The validation results indicate that the proposed exponential model is superior to the LBL and MSC methods for compensating turbidity, rendering it suitable for use in online spectrometers to enhance COD measurement accuracy in turbid water. The light scattering caused by particles in water is influenced by various factors, such as the concentration, diameter, and refractive index of the particle, as well as the measuring angle and wavelength of incident light (Huber and Frost, 1998). It is important to acknowledge that these factors may also impact the turbidity compensation model, thus necessitating recalibration of the model in response to changes in application conditions.

## 4. Conclusion

Presently, the presence of turbidity in water poses a significant challenge for accurate COD measurements based on UV-vis spectrometry. Therefore, implementing turbidity compensation techniques is necessary to ensure data validation and precision. However, current methods for such compensation are too intricate to be automated for online spectrometers. Herein, an exponential model with two parameters was established for turbidity compensation. One parameter was kept constant, while the other was varied quadratically with turbidity. This simple model satisfies the requirements of embedded programming and local calibration of online sensors. Based on the model, the equivalent turbidity absorbance at each wavelength can be obtained. Compensation across the entire spectrum is then achieved by subtracting the equivalent absorbance to obtain the COD-specific absorbance. Finally, accurate detection of COD can be achieved using the compensated spectra. Compared with existing models, such as the LBL and MSC models, the exponential model demonstrates significantly superior performance. Although its performance in the UV region is unsatisfactory owing to filtration procedures, the consistency between the compensated spectra and filtered wastewater samples in the visible range suggests that the exponential model remains applicable for natural water analysis. In summary, the exponential model is demonstrated to be a highly effective approach for enhancing the precision of COD measurement by online spectrometers, offering valuable support for compensation techniques aimed at eliminating turbidity interference.

## Data availability statement

The original contributions presented in the study are included in the article/Supplementary material, further inquiries can be directed to the corresponding author.

## Author contributions

HW, HX, and TX performing all the experiments and writing the manuscript. JF and JZ drawing and summarizing figures. XL designing, managing the project, and editing. HW, HX, TX, JF, JZ, and XL discuss the results and commented on the manuscript. All authors contributed to the article and approved the submitted version.

## Funding

This work was supported by the doctoral research foundation of Hubei university of Science and Technology (BK202321) and Major Project of Scientific and Technological Innovation in Hubei (Grant No. 2020BGC028, 2019AAA057).

## Conflict of interest

The authors declare that the research was conducted in the absence of any commercial or financial relationships that could be construed as a potential conflict of interest.
